# A decade of contributions to understanding and ameliorating attention deficit-hyperactivity disorder

**DOI:** 10.1186/s12993-016-0097-9

**Published:** 2016-04-06

**Authors:** Peter R. Killeen

**Affiliations:** Department of Psychology, Arizona State University, Tempe, AZ USA

**Keywords:** Attention deficit-hyperactivity disorder, Decade, Retrospect

## Abstract

**Background:**

It has been 10 years since Terje Sagvolden founded *Behavioral and Brain Functions.*

**Body:**

After setting the context, this paper reviews some of the contributions of articles in the journal to the literature on ADHD over that decade. Those articles provide a cross section of some of the most important ongoing themes in ADHD research.

**Conclusion:**

Terje’s faith in the new journal was well founded. It has survived the first threat of new journals, crib death, because of the continuing quality and relevance of the articles it carries. It has a diversified portfolio of similar research in many other fields of related interest.

## Background

Myth pictures the Vikings as raiders and barbarians. During the three centuries of their northern dominance, however, the majority of the Norsemen’s energy was spent as traders and settlers, connecting cultures from Newfoundland to Russia, from Iceland to Marseilles and Pisa. For this they crafted their trademark vehicle, the longboat.

Terje Sagvolden was a Norwegian who grew up near a recovered Viking longboat and a restored Viking village around Oslo. Like his forbearers he had an itch to travel, starting with a post-doc in America with Charles Catania, where he came to appreciate the power of behavioral analyses. In the early years of this century he became interested in attention deficit-hyperactivity disorder (ADHD), and began to study it from a behavioral perspective. To do this effectively he realized he would need a vehicle, and he crafted two of them. The first was a niche in an Oslo mansion that housed the *Institute for Advanced Study* where he spent a year with a cadre of researchers working on ADHD; the second was his trademark vehicle, the journal *Behavioral and Brain Functions* (BBF).

I was a fellow at the institute thanks to Terje’s enterprise, along with many experts who voyaged from spots on the globe far beyond the ken of the Vikings. This was the year that Terje founded BBF, and I remember him accosting me, with his irrepressible enthusiasm, about this new journal, its goals, name, logo and aspirations. The journal has lived up to the aspirations, even though like most adventurers, Terje did not live to see the enduring success of his foundling. Nor did he see how this journal, dedicated to research which “provides insight on the neurobiological mechanisms underlying behavior and brain function and dysfunction”, would attract a much broader field of investigations than on just ADHD. It did that from the start, with almost half of the articles concerned with ADHD in the first 2 years, decreasing to a quarter in recent years, even while the total number published over that time has more than doubled. These articles have contributed importantly to our understanding of ADHD. Here are a few of the highlights of that subset of articles, chronologically encountered.

## Highlights

The administration of low oral doses of methylphenidate (MPH) to rats affects pre-frontal cortical functions in ways similar to the effects seen in humans, both typically-developing and those with ADHD [1]. This similarity of mechanism endorses rats as a viable model for the study of those neural mechanisms that underlie the therapeutic effects of MPH on humans. Both noradrenergic α2 adrenoceptor and dopamine D1 receptor stimulation were seen to contribute to the cognitive-enhancing effects of MPH. Cited by three hundred other papers, this research provided strong support for the ensuing study of rat models of ADHD, such as that of Russell and colleagues [2], who further characterized the noradrenergic and dopaminergic abnormalities found in several animal models of ADHD. But even then, BBF wasn’t all about rats. Ever wonder why there are such extensive comorbidities among neurobiological disorders? Many involve problems with the monoamines, and irregularities in the COMT gene, which affects the degradation of monoamines such as dopamine, co-occur in many of these disorders, including some cases of ADHD [3, 4]. Another study in the birth year of BBF found that ADHD made poorer executive decisions on the Iowa Gambling Task, and that the difference from controls could not be accounted for by differences in working memory or intellectual ability [5].

It is not only decision-making that is impaired in ADHD. The cerebellum, which, among its many functions, controls fine motor skills, is differentially affected in ADHD. Therefore motor skills should be impaired. In an adventurous study of seven ethnic groups in Africa, Meyer and Sagvolden [6] showed substantial differences in several motor tasks between ADHD and controls (as did Stray and colleagues [7] in tamer subjects). Such research may help to bring other dimensions than observer reports to bear in the diagnosis of this disorder. The culprit in ADHD is often thought to be poor dopamine regulation. Russell and associates [8], however, posited that ADHD might primarily concern energy management, not dopamine management. Another monoamine, noradrenaline (NA), plays a key role in stimulating parts of the brain to release energy to feed rapidly firing neurons. Without that food, firing would slow and response times would become slower and more erratic. Recent genetic analysis [9] also supports the noradrenergic hypothesis for the pathophysiology of ADHD. If that is the case, high frequency EEG oscillations would be damped, as energy supply is a rate-limiting factor. This prediction was recently validated in this journal [10].

Conduct disorder is a frequent sequela of ADHD, and Beaver and colleagues [11] found that it was associated with gene × gene interactions between DRD2 and DRD4. This is noteworthy because these are part of the inhibitory family of dopamine (as opposed to the D1–D5 family—the positive cousins). Similar interactions were found in an elegant study by Eisenberg and colleagues [12], who showed substantial genetic effects on the rate of discounting future monetary rewards. But ADHD is not just about dopamine and noradrenaline; that other monoamine, serotonin, also plays a role in the syndrome. Genetic research typically requires large samples—of both subjects and scientists—and the village led by Oades [13] analyzed twelve-hundred individuals to show how genes associated with serotonin modulate impulsive behavior and cognition. If you wish to learn more about genetics and ADHD, and the methods and modes of interpretation of these complex data by leading researchers in this field, then you must read the article by Kunsti and associates [14].

An important theory of ADHD—the *dynamic developmental theory*—assumed aberrant reinforcement processes to be an important underlying endophenotype of that condition. Recent research [15] provides some support for that hypothesis; and other research shows that such reinforcement dysfunction may in some cases be meliorated by polyunsaturated fatty acids [16]; so eat more fish! Many of the participants from the Center conclave reconvened to review the evidence and rationale for the reinforcement hypothesis, providing in [17] one of the more accessible accounts of the contemporary understanding of reinforcement processes of relevance to that theory, and to ADHD.

Animal models played a very large role in the genesis of the dynamic developmental theory, as they do for research on neurobehavioral disorders in general. But what is an animal model? Anyone contemplating such research, either as consumers or creators of it, should first read the article by van der Staay and colleagues [18], who lay out an excellent framework for developing animal models and assessing their quality. Drugs also play a very large role in research on neurobehavioral disorders; but what about prodrugs? Just what, you may ask, are prodrugs? They are substances which, when consumed, are converted into an active agent. Lisdexamfetamine dimesylate (LDX) is a prodrug that the body converts into d-amphetamine. In a randomized double-blind placebo-controlled crossover study, Wigal and associates [19] showed it to be as least efficacious as methylphenidate (MPH) in a simulated adult workplace. Subsequent work has proved its effectiveness in other contexts, and also that it has somewhat lower abuse potential than d-amphetamine.

Another theory of ADHD is that it is all about inhibition failure. McLoughlin and associates [20] provide evidence that there is no core response inhibition deficit in ADHD. Their results in adults replicate those in children, suggesting continuity of the same underlying abnormal processes; but not ones that should be thought of as inhibitory.

Early trauma, as fetus or child, is one of the important causal factors in the genesis of ADHD. Sterley and colleagues [21] found that the standard rat model of ADHD (the SHR) also responded to early trauma (maternal separation) in significantly different ways than its control strain. Early stress can also decrease spatial working memory later in life [22]. How does early experience modulate gene expression this way? Through epigenetic processes such as DNA methylation, a process affected by polymorphisms of the kind associated with ADHD [23].

An important criterion for animal models is that they respond in ways similar to the human presentations for which they serve as model, both in behavioral tests and in response to relevant drugs. There has been ambiguity in the literature on the effects of methylphenidate (MPH) on rat models such as SHR. Cao and colleagues [24] provide important evidence that the SHR can satisfy that criterion. Not only were SHR poorer than controls on discrimination and reversal learning and set shifting, moderate doses of MPH significantly improved performance on set shifting. Other research showed differential sensitization to MPH in SHR [25].

## Conclusions

The above highlights reflect both my own interests, and also my criterion that most of the articles mentioned have accrued more than ten citations per year every year since they went to press. This is an estimable record, and speaks to the hard work of the editors and reviewers in maintaining the quality that Terje set as a standard. BBF is the name of a longboat that plies the ether, carrying ideas and techniques to every part of the globe. It brings an impressive range of articles, ones involving clinically sophisticated analyses of comorbidities, executive functions, and endophenotypes; and methodologies including genetics, neuroimaging, neuropharmacology; and animal models, across species and across lifespans, on topics of current interest to the field. It enriches not only the lives of those who trade with it, but all in the larger world whose lives they improve with their work.

## Abbreviations

ADHD: attention deficit-hyperactivity disorder (a category of developmental disability)
AKA: “also known as” (an acronym indicating a synonym)
BBF: Behavioral and Brain Functions (this journal)
COMT: catechol-*o*-methyltransferase (an enzyme that degrades monoamines such as dopamine and norepinephrine)
DNA: deoxyribonucleic acid (a molecule that carries most of the genetic information)
DRD2[4]: genes that code for dopamine receptor D2 [or D4]. (D2 is the main receptor for antipsychotic drugs)
EEG: electroencephalography (electrodes placed atop the scalp yield signals analyzed with this technique)
MPH: methylphenidate (a monoamine reuptake inhibitor, aka Ritalin^®^)
NA: noradrenaline (aka norepinephrine, a neurotransmitter)
LDX: lisdexamfetamine dimesylate (a prodrug that the body converts to d-amphetamine)
SHR: spontaneously hypertensive rat (an animal model of ADHD)
WKY: Wistar-Kyoto rat (a control animal for the SHR)
